# Editorial: Neuromuscular and biomechanical alterations in chronic ankle instability

**DOI:** 10.3389/fspor.2025.1719502

**Published:** 2025-10-28

**Authors:** Kevin Deschamps, Luciana Labanca, Giovanni Matricali

**Affiliations:** ^1^ Department of Rehabilitation Sciences- Musculoskeletal Rehabilitation Research Group, KU Leuven, Brugge, Belgium; ^2^ Department of Orthopaedics, University Hospitals Leuven, Leuven, Belgium; ^3^ Institute of Orthopaedic Research and Training, KU Leuven, Leuven, Belgium; ^4^ Rizzoli Orthopaedic Institute, Bologna, Italy

**Keywords:** chronic ankle instability, foot biomechancis, pathomechanical insufficiencies, sensory-perceptual impairments, motor-behavioral alterations

**Editorial on the Research Topic**
Neuromuscular and biomechanical alterations in chronic ankle instability

## Introduction

Chronic ankle instability (CAI) is one of the most common long-term sequelae of lateral ankle sprains, which themselves represent the most frequent musculoskeletal injury in sport and physical activity (1)*.* Epidemiological evidence suggests that up to 40% of individuals who sustain a first-time lateral ankle sprain will go on to develop CAI, characterized by recurrent sprains, frequent “giving-way” episodes, and persistent symptoms such as pain, swelling, weakness, and reduced function (2, 3)*.*

Recent conceptual advances, particularly the updated model proposed by Hertel and colleagues, highlight CAI as a multifactorial condition resulting from the interaction of pathomechanical insufficiencies (e.g., ligament laxity and arthrokinematic restrictions), sensory-perceptual impairments (e.g., proprioceptive errors, altered somatosensation, pain and kinesiophobia), and motor-behavioral alterations (e.g., delayed neuromuscular responses and abnormal gait and landing mechanics) (4). This framework is situated within a biopsychosocial model, recognizing that personal and environmental factors, along with self-organization and perception–action cycles, contribute to each patient's unique clinical outcome. Importantly, this heterogeneity underscores the need for individualized assessment and rehabilitation strategies.

The Research Topic *Neuromuscular and Biomechanical Alterations in Chronic Ankle Instability* was launched to advance understanding of these mechanisms, bridge the gap between laboratory findings and clinical applications, and stimulate novel approaches to prevention and rehabilitation. The **five articles** in this Research Topic offer diverse and complementary perspectives—ranging from mechanistic insights into sensorimotor control to biomechanical assessments of functional movements and applied strategies for rehabilitation. Collectively, they underscore the importance of a multidimensional approach to CAI that integrates neurophysiology, biomechanics, and clinical practice.

## The Research Topic articles

In their study, Chang et al. examined the effects of combining acupuncture with strength training in college students with chronic ankle instability. Over an eight-week program, participants receiving both interventions demonstrated greater improvements in balance, muscle strength, and ankle kinesthetic sensation compared to those performing strength training alone. While both groups improved CAIT scores and ankle function, the addition of acupuncture enhanced dorsiflexion and plantar flexion strength as well as anterior–posterior balance control. The authors attribute these benefits to acupuncture's role in promoting circulation, neural regulation, and tissue repair. These findings suggest that integrating acupuncture with conventional rehabilitation may offer a more comprehensive and effective strategy for managing CAI.

Shao et al. introduced Ankle Inversion Discrimination Apparatus-Walking (AIDAW) as a novel tool for assessing ankle proprioception during walking in individuals with chronic ankle instability (CAI). The device demonstrated good test–retest reliability and effectively distinguished between CAI and healthy participants. AIDAW scores were moderately correlated with both the Y Balance Test and the Cumberland Ankle Instability Tool, linking proprioceptive discrimination to functional balance and perceived instability. By providing ecologically valid assessments of ankle function during walking, AIDAW seems to address a critical gap in current practice. These findings highlight its potential for monitoring rehabilitation outcomes and guiding interventions that target proprioceptive deficits in CAI.

Altered balance and gait is another hallmark of CAI. Decker et al. assessed whether individuals with chronic ankle instability differ from uninjured controls in their ability to maintain balance and adapt gait when exposed to progressively challenging sensory perturbations using the Sensory Organization Test (SOT) and the Locomotor Sensory Organization Test (LSOT). The findings seem to point towards adaptation to sensory challenges in a manner similar to uninjured peers, suggesting that standard SOT and LSOT protocols may not be sensitive enough to capture CAI-specific deficits. This highlights the need for more demanding, ecologically valid balance and gait assessments to advance both research and clinical evaluation of CAI.

In the fourth article, Hong et al. examined how dance training and chronic ankle instability (CAI) affect postural control and visual reliance. Individuals with CAI showed poorer static balance, particularly without visual input, while dancers—regardless of CAI status—demonstrated greater reliance on vision than non-dancers. No significant group differences were observed in dynamic postural control during landing tasks. The findings suggest that dance training may foster compensatory strategies based on vision, which can become a limitation when visual feedback is absent. The authors recommend that assessments of postural control should distinguish between somatosensory dependence and visual reliance.

Finally, the study of Peters-Dickie et al. investigated how a history of lateral ankle sprains (LAS) and CAI influence gait biomechanics in runners. While individuals with CAI and acute LAS reported greater perceived instability and reduced self-reported function, traditional biomechanical analyses at comfortable running speeds did not reveal significant group differences, suggesting adaptive strategies that mask deficits. In contrast, walking assessments highlighted altered mechanical work recovery in copers, pointing to subtle compensatory mechanisms. The findings emphasize the importance of integrating self-reported outcomes into clinical assessments, as subjective deficits may not be detected through standard biomechanical testing. Clinically, the results support the safe reintroduction of running during rehabilitation while also underscoring the need for more sensitive and challenging biomechanical assessments to uncover hidden impairments.

## Concluding comments

The articles collected within this Research Topic underscore the multifactorial and heterogeneous nature of chronic ankle instability. By examining novel rehabilitation strategies, advancing proprioceptive assessment tools, and characterizing adaptations in balance and gait across different populations, these contributions collectively extend the current understanding of neuromechanical alterations associated with the condition. A recurring theme across the studies is the importance of ecologically valid assessments and the integration of patient-reported outcomes to capture deficits that may not be evident through traditional measures. These insights highlight the need for individualized and multidimensional rehabilitation approaches that address both central and peripheral mechanisms of dysfunction. To achieve this, multidisciplinary collaboration of (para-)medical specialists in rehabilitation, physiotherapy, orthopedics, human movement science, and social sciences is mandatory. Therefore, future progress will depend on sustained interdisciplinary collaboration, with the aim of translating mechanistic knowledge into more effective strategies to prevent reinjury, optimize function, and improve long-term health outcomes in individuals with chronic ankle instability.
